# Correction: Shahbazi et al. Effective Low-Energy Hamiltonians and Unconventional Landau-Level Spectrum of Monolayer C_3_N. *Nanomaterials* **2022**, *12*, 4375

**DOI:** 10.3390/nano15211678

**Published:** 2025-11-05

**Authors:** Mohsen Shahbazi, Jamal Davoodi, Arash Boochani, Hadi Khanjani, Andor Kormányos

**Affiliations:** 1 Department of Physics, Faculty of Science, University of Zanjan, Zanjan P.O. Box 45195-313, Iran; jdavoodi@znu.ac.ir; 2 Department of Physics, Kermanshah Branch, Islamic Azad University, Kermanshah P.O. Box 671791-7855, Iran; arash_bch@yahoo.com; 3 Quantum Technological Research Center (QTRC), Science and Research Branch, Islamic Azad University, Tehran P.O.Box 14515-755, Iran; 4 Department of Physics, University of Tehran, Tehran P.O. Box 14395-547, Iran; hadikhanjani@gmail.com; 5 Department of Physics of Complex Systems, Eötvös Loránd University, 1117 Budapest, Hungary

In our published paper [1], we have identified misprints and errors in the values of the k·p model parameters, which were obtained by fitting the results of the density function theory (DFT) band structure calculations.

## 1. Effective Mass Values in Table 1

The corrected effective masses in Table 1 are as follows:

## 2. Corrected k·p Model Parameters

The effective mass errors propagated to the derived k·p model parameters. The corrected parameters, which appear in $H_{k\cdot p}^{\Gamma }$ and $H_{k\cdot p}^{M}$ in Equation (3) and Equation (6), respectively, are as follows:

$$\begin{array}{cccccc} \alpha_{1} & =51.18, & \alpha_{2} & =17.68, & \alpha_{3} & =13.89\\ \beta_{1} & =-111.7, & \beta_{2} & =-0.71, & \beta_{3} & =125.3\\ \gamma_{21} & =5.61. \end{array}$$

The parameters *α*_1_, *α*_2_, *α*_3_, *β*_1_, *β*_2_, and *β*_3_ are in units of eVÅ^2^, and *γ*_21_ is in units of eVÅ.

## 3. Corrections to Landau-Level Plots (Figures 4 and 5)

As a result of the correction to the values of $\alpha_{2}$ and $\alpha_{3}$, the Landau-level plots shown in Figure 4 and Figure 5 have slightly changed compared to those originally published (see Figure 4 and Figure 5). The conclusions with regards to the properties of the Landau-level spectrum are not affected.

## 4. Corrections to Wigner–Seitz Radius Mentioned in Section 5.1

The change in the effective masses affects the dimensionless Wigner–Seitz radius $r_{s}$. This is important because we used $r_{s}$ to assess the possible relevance of the electron–electron interactions on the Landau level spectrum. The effective mass values for the conduction bands CB and CB+1 given in the original manuscript were significantly heavier than the corrected values shown in Table 1. Therefore, one may expect that the electron–electron interactions are less important than originally anticipated because of the larger kinetic energy (smaller effective mass) of the charge carriers.

The Wigner–Seitz radius is given by $r_{s}=1/\left(\sqrt{\pi n_{e}}a_{B}^{*}\right)$, where $n_{e}$ is the electron density, $a_{B}^{*}=a_{B}\left(\kappa m_{e}/m^{*}\right)$ is the effective Bohr radius, $m^{*}$ is the effective mass, κ is the dielectric constant, and $a_{B}$ is the Bohr radius. Taking κ=5 and an electron density of $n=4\cdot 10^{12}$ ${\mathrm{cm}}^{-2}$, one finds $r_{s}=2.35$ for the heavier conduction band, where $m^{*}/m_{e}=0.22$.

## 5. Corrections to Appendix A

There was a typo in Equation (A2), which shows one of the partners of the wave function that transform according to the $E_{1g}$ irreducible representation of the point group $D_{6h}$. The correct form of wave function $\phi_{4}$ reads as follows:

$$\begin{array}{c} \phi_{4}=\frac{1}{\sqrt{6}}\left[p_{Z}^{1,C}+\Omega^{5}p_{z}^{2,C}+\Omega^{4}p_{z}^{3,C}-p_{z}^{4,C}+\Omega^{2}p_{z}^{5,C}+\Omega p_{z}^{6,C}\right]. \end{array}$$

## Figures and Tables

**Figure 4:**
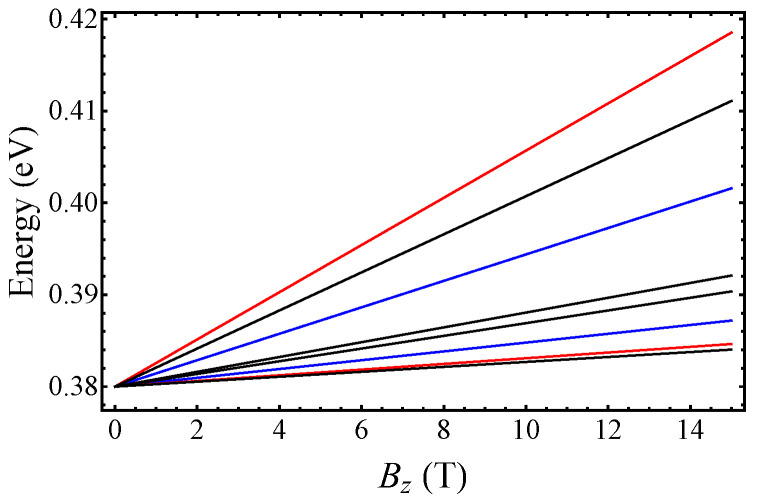
Landau levels in the CB at the Γ point of the BZ as a function of the out-of-plane magnetic field $B_{z}$. Blue lines show $E_{0}^{\Gamma }$ and $E_{1}^{\Gamma }$, given below in Equation (15), and red lines indicate the first two LLs that can be obtained from the Ansatz in Equation (16). Black lines show the “conventional” LLs. See the manuscript.

**Figure 5:**
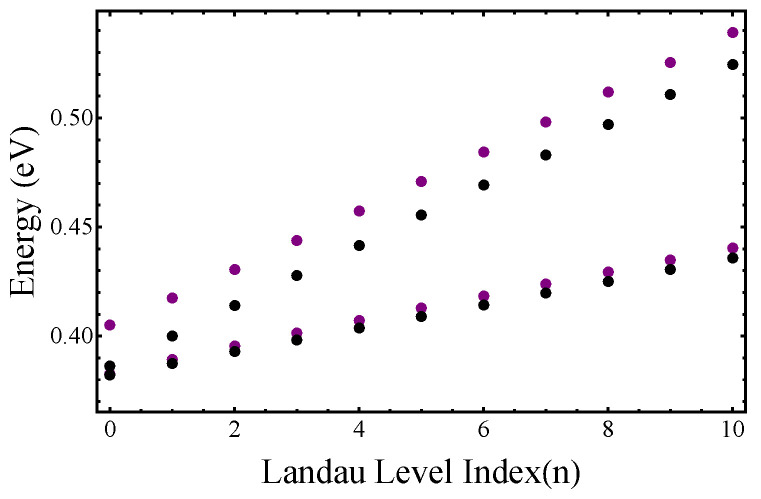
Landau levels in the CB at the Γ point of the BZ as a function of the LL index n for $B_{z}=10$ T. Magenta dots indicate LLs that can be obtained from the Ansatz in Equation (16) of the manuscript, and black dots show the “conventional” LLs. See the manuscript.

**Table 1:** Effective masses at the Γ and *M* points.

	All Directions	*M*–Γ Line	*M*–K Line
$m_{vb}^{\Gamma }/m_{e}$	0.07	-	-
$m_{cb}^{\Gamma }/m_{e}$	0.22	-	-
$m_{cb+1}^{\Gamma }/m_{e}$	0.08	-	-
$m_{vb}^{M}/m_{e}$	-	−0.25	−0.03
$m_{cb}^{M}/m_{e}$	-	−0.19	0.03
